# Femtosecond dynamics of energetic electrons in high intensity laser-matter interactions

**DOI:** 10.1038/srep35000

**Published:** 2016-10-07

**Authors:** R. Pompili, M. P. Anania, F. Bisesto, M. Botton, M. Castellano, E. Chiadroni, A. Cianchi, A. Curcio, M. Ferrario, M. Galletti, Z. Henis, M. Petrarca, E. Schleifer, A. Zigler

**Affiliations:** 1 Laboratori Nazionali di Frascati, 00044 Frascati, Italy; 2 Racah Institute of Physics, Hebrew University, 91904 Jerusalem, Israel; 3 University of Rome Tor Vergata, 00133 Rome, Italy; 4 University of Rome Sapienza, 00185 Rome, Italy

## Abstract

Highly energetic electrons are generated at the early phases of the interaction of short-pulse high-intensity lasers with solid targets. These escaping particles are identified as the essential core of picosecond-scale phenomena such as laser-based acceleration, surface manipulation, generation of intense magnetic fields and electromagnetic pulses. Increasing the number of the escaping electrons facilitate the late time processes in all cases. Up to now only indirect evidences of these important forerunners have been recorded, thus no detailed study of the governing mechanisms was possible. Here we report, for the first time, direct time-dependent measurements of energetic electrons ejected from solid targets by the interaction with a short-pulse high-intensity laser. We measured electron bunches up to 7 nanocoulombs charge, picosecond duration and 12 megaelectronvolts energy. Our ’snapshots’ capture their evolution with an unprecedented temporal resolution, demonstrat- ing a significant boost in charge and energy of escaping electrons when increasing the geometrical target curvature. These results pave the way toward significant improvement in laser acceleration of ions using shaped targets allowing the future development of small scale laser-ion accelerators.

Recent advances in laser technology opened up new horizons in modest-scale experiments of sub-picosecond light-matter interactions, enabling new research areas like astrophysics in laboratory^1^, high energy density experiments^2^ and novel schemes for particle acceleration^3^,^4^. Ion acceleration from thin foils irradiated by high-intensity short-pulse lasers, in particular, has attracted high attention during the past decade since the emitted ion and proton pulses contain a large amount of particles with energies in multi-MeV range^5^,^6^,^7^ and are tightly confined in time (picosecond-scale) and space (source radius is few microns). These outstanding characteristics provide possibilities for a wide range of applications in nuclear and medical physics^8^.

The physical picture of the process is the following. Electron jets are produced at the early stages of the interaction^9^. Some electrons are energetic enough to escape the target while others remain at the vicinity of the surface, re-hitting it and ejecting secondary electrons^10^. After the escaping of the first ones, a positive unbalanced charge is left on target, leading to the formation of the electrostatic potential that in turn governs the ion acceleration^11^,^12^. The typical timescale of such phenomena is on the sub-picosecond level. During this process the electronic cloud locked near the target is thermalized and there are energetic electrons (on the ‘hot’ tails of the overall energy distribution) that can still escape from the target. This process however comes to an end when their energy can not overcome the electrostatic potential induced near the target surface, whereas a second slower expansion-relaxation process takes over^13^. Although the plasma density generated away from the target drops by orders of magnitude, the majority of electrons is confined within a distance of the order of the Debye length^14^.The escaping energetic electrons constitute the electric current charging positively the target^15^ and leading to the generation of a potential barrier. Its lifetime is dictated mainly by the return currents, cloud dynamics and thermalization rates at the target surface. The intensity and time duration of this barrier eventually sets the limit on the late-time processes e.g. acceleration of the positively charged ions^16^. For sub-picosecond laser pulse irradiation, one can neglect the charge neutralization of the positively charged ions by the electrons coming from outer darkened sections of the target and assume that the number of escaped energetic electrons defines the net positive charge left on the target surface^13^. The subsequent cooling process, including multiple collisions with the surrounding ions, sets the maximal time of the target charging, i.e. the effective lifetime of the potential barrier. A direct experimental evidence of these processes requires sub-picosecond measurements of charge density near the surface or alternatively tracing down the escaping electrons. So far this task remained elusive and only indirect time integrated measurement of radiated electromagnetic pulses^17^,^18^ or magnetic fields^19^ were reported.

In the following we provide temporally resolved measurements of energetic electrons ejected from solid targets during the interaction with a short-pulse high-intensity laser. We measured the total charge and the temporal profile of electron pulses with up to 7 nC charge and 12 MeV energy with picosecond duration. The results show that, when using high-power ultra-short laser pulses focused on different target geometries (namely planar, wedged and tip shapes), there is a significant increase in the charge and energy of the escaping electrons when the geometrical target curvature is increased. It represents a direct evidence of the growth of the electrostatic potential induced near the target surface and thus an enhancement of the accelerating gradient for the emitted ions.

## Setup of the experiment

**Figure 1:**
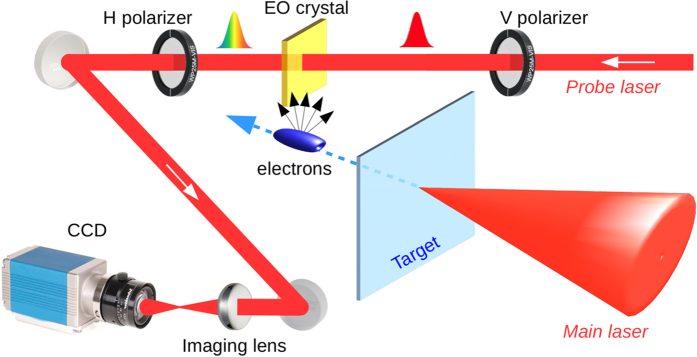
Sketch of the experiment. An *f*/10 parabola focuses the main laser on a metallic target ejecting a cloud of energetic electrons. An electro-optic crystal (ZnTe) is located 1 mm downstream the target. The Coulomb fields of the moving electrons optically modify the crystal, making it birefringent. This changing is temporally encoded by a linearly polarized probe laser. By measuring the polarization modulation of the probe laser, the main properties of the emitted electrons (charge, energy, temporal profile) are retrieved.

The experiment, depicted in Fig. 1, has been performed with the FLAME laser at the SPARC_LAB test-facility^20^ by focusing its high-intensity ultra-short-pulses (up to 4 J energy and 35 fs pulse duration) on solid targets of different thicknesses and shapes. Snapshots of the emitted electrons are provided by an Electro-Optical Sampling (EOS) device^21^, a temporal diagnostics commonly used in accelerator facilities^22^,^23^, able to provide single-shot and non-destructive measurements for the longitudinal profile of charged particle beams. The EOS system we employed makes use of a 500 *μm*-thick ZnTe electro-optic crystal installed 1 mm downstream the target. Being this distance much larger than the Debye length (less than 1 *μm* in our experimental conditions), only the highly energetic ejected electrons that escape the potential barrier are able to reach that location. A probe laser (35 fs duration), directly split from the main laser, illuminates the crystal while simultaneously the electron cloud is moving below it. Such ultra-short probe laser allows to achieve less than 100 fs as temporal resolution, mainly limited by the implemented electro-optic crystal^24^. The high resolution EOS diagnostic technique allows us to operate on the same time scale of the process, determined by the duration of the driving laser pulse^10^.

The EOS diagnostics exploits the large electric fields associated to relativistic charged particles. When they move near the electro-optic crystal, their Coulomb field *E*_*b*_(*t*) makes the crystal birefringent. As a consequence, if a linearly polarized probe laser simultaneously passes through the crystal, its polarization is rotated by an angle Γ(*t*) ∝ *r*_41_*E*_*b*_(*t*)*d*, where *d* and *r*_41_ are the crystal thickness and electro-optic coefficient, respectively. The probe polarization is thus modulated according to the electron bunch temporal profile. Our EOS system exploits a probe laser directly split from the main laser, ensuring a jitter-free synchronization, and implements the spatial encoding technique^25^ in which the bunch longitudinal profile is encoded along the probe transverse profile. The encoding is obtained when the probe laser crosses the crystal with an angle, that in our case is *θ*_*i*_ = 28°. In such way the bunch longitudinal coordinate *t*_*i*_ is related to the laser transverse one *x*_*i*_ by the relation *t*_*i*_ = *x*_*i*_ tan *θ*_*i*_/*c*, with *c* the vacuum speed of light. Being 6 mm the diameter of the probe laser, it follows that the resulting active time window provided by the EOS is about 10 ps. The process ends by converting the induced modulation in the probe polarization in a modulation in its intensity (readable by a CCD camera) by means of a linear polarizer installed downstream the EOS crystal, whose optical axis is rotated by 90 with respect to the initial probe laser polarization. More details about the laser system and the implemented EOS diagnostics are presented in sec. [methods]Methods.

## Results

Previous reports of ion acceleration by high intensity short pulse lasers have demonstrated a significant energy enhancement of the accelerated ion when structured targets^26^,^27^,^28^ were used instead of the conventional planar target in the Target Normal Sheath Acceleration (TNSA) scheme. The underlying interpretation of these results is that, during the interaction of the laser pulse with sharp structured targets, higher quantity of electrons escape the target leaving behind a stronger potential well, which in turn can accelerate the ions to higher energies. In order to prove this conjecture a direct time-resolved measurement of the escaping electrons is required. We therefore employed our method and investigated the influence of target shape on the amount and energy of the escaping electrons by using a 10 *μm*-thick aluminum foil, a wedged shape of stainless steel razor blade and a tip shape of a needle. Measuring the charge quantity and energy of the escaping electrons by means of the EOS detector provides the required evidence for the field-enhancement conjecture. The energy information, in particular, is estimated by exploiting the single-shot feature of the EOS system, allowing to use it as a time of flight monitor^25^.

**Figure 2:**
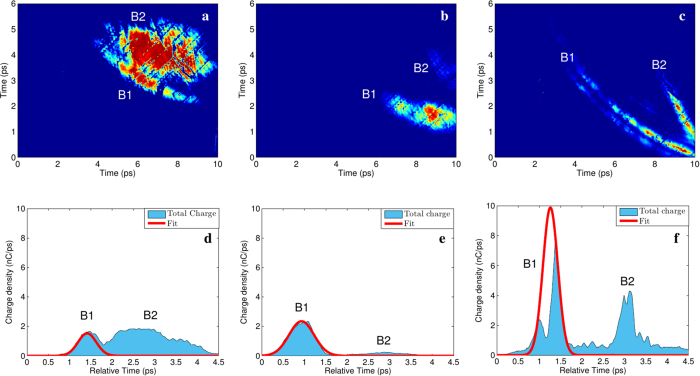
Snapshots with different target shapes. Signatures of the escaping electrons from (**a**) planar, (**b**) wedged and (**c**) tipped targets. The emitted charges are, respectively, (**a**) 1.2 nC (B1) and 3 nC (B2); (**b**) 2 nC (B1) and 0.3 nC (B2); (**c**) 7 nC (B1) and 3 nC (B2). The gaussian envelopes represent the extrapolated charge profiles of each bunch. (**d–f**) Corresponding longitudinal charge profiles. A 10^2^ neutral density filter has been used in (**b**,**c**) to avoid saturation of the CCD camera.

The results, summarized in Fig. 2 have been obtained by focusing the FLAME laser on different target shapes. The geometry of our EOS setup (the bunch is moving below the crystal and normally to it while the probe laser propagates laterally from right to left) determines the curved shape of the retrieved signals. The snapshots show that the escaping energetic electrons from the planar and blade targets present a secondary, broadened temporal structure (see Fig. 2(d,e), respectively). The duration and energy of the electron bunch is derived by measuring the bunch time of flight up to the EOS detector and by fitting it with numerical EOS simulations. In the case of the planar foil target, the resulting snapshot in Fig. 2(a) shows the presence of a first emitted bunch with approximately 1.2 nC charge and 7 MeV energy followed by a second broadened structure carrying a larger amount of particles (about 3 nC). If we assume that the delay (about 1.5 ps, see Fig. 2(d)) is due to different bunch velocities, the latter one has about 1 MeV energy. For the wedged target, the snapshot in Fig. 2(b) shows a similar structure. The first bunch now carries a larger amount of electrons (2 nC) at the same energy (7 MeV) while the charge in the second bunch is strongly reduced to 0.3 nC. The temporal delay in this case is about 2 ps, as reported in Fig. 2(e). Electron bunches coming from the tip target are shown in Fig. 2(c). In this case the interaction with laser produced a much larger number of released electrons (about 7 nC) at higher energies (about 12 MeV). Due to the large amount of charge, the birefringence induced in the ZnTe crystal leads to a rotation of the probe laser polarization larger than *π*/2 and the EOS signal in Fig. 2(f) is consequently distorted. The overlaying red line shows the retrieved charge profile. These results provide a direct evidence of charge and energy boost when using sharp tips. Another feature, possibly attributed to this target shape, consists in the presence of a second smaller bunch (B2), carrying about 3 nC charge. The difference in the slope of the two signals in Fig. 2(c) may be due to the fact that B2 is emitted along a different path, rotated by ≈50 with respect to B1. It follows that B2 traveled for a longer distance (about 600 *μm*) and its signal is delayed by about 2 ps, as reported in Fig. 2(f).

### Particle-In-Cell Simulations

**Figure 3:**
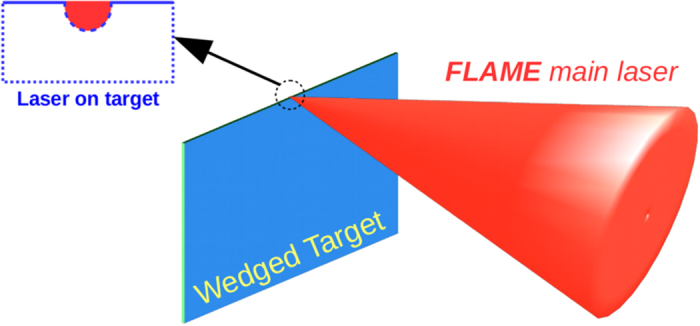
Geometry of the laser-wedged target interaction. The main laser is focused on the tip of the wedged target. A detailed view of how the laser irradiates the target surface is shown on the top-left corner.

The experimental results shown in Fig. 2 are in agreement with Particle-In-Cell (PIC) simulations. We conducted a numerical study in order to reproduce the interaction of a high-intensity short-pulse laser with wedged targets by using the 2D particle-in-cell (PIC) code TURBOWAVE^29^. The sketch of the interaction is reported in Fig. 3. Numerical simulations of the reported experiment include detailed description of the interaction near the surface and reveal the formation of the electron cloud and ejection of the fast energetic electrons. The simulations consist in a surface with micron-scale target local perturbations (consistent with electron scanning microscope images of the blade material used in the experiment) interacting with laser intensity of 10^18^ W cm^−2^, a spot diameter of 10 *μm* and an overall 30 fs duration. The simulation region is 40 × 40 *μm*^2^ with 10^−2^ *μm* cell size in order to reproduce the surface roughness. Considering the pre-pulse effect on the target, the blade is considered as a Fe^5+^ plasma and each cell of the plasma region contains initially 512 particles. This resolution enables a proper description of the expected high gradients in the density and generated potential.

**Figure 4:**
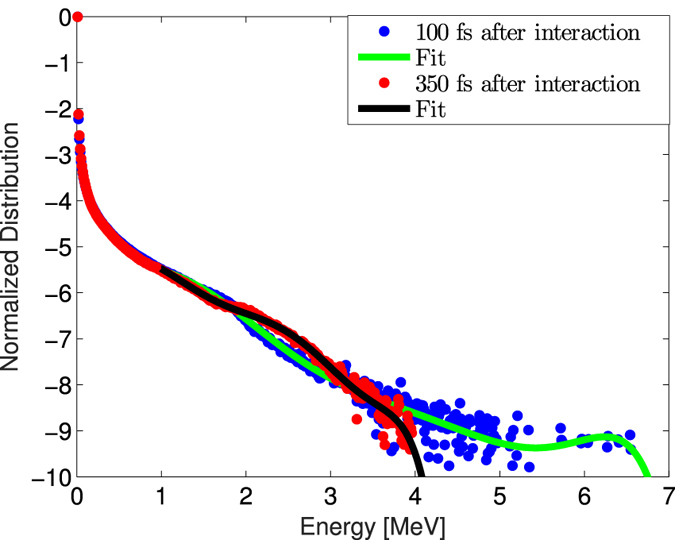
Energy spectrum of the emitted electrons. The blue (red) points have been obtained 100 fs (350 fs) after the interaction with the laser. The solid lines represent the computed fit on such distributions. The *y*-axis is in logarithmic scale.

Figure 4 shows the electron spectrum as function of energy obtained by placing a virtual screen in proximity of the target. The blue (red) points represent the electron distribution 100 fs (350 fs) after the laser hits the target surface. The semilog plot demonstrates that the majority of the electrons are slow (below 3 MeV) because they continue to stay near the target while the time increases. There is also a smaller component of trapped electrons up to about 4 MeV energy. On the contrary, electrons with higher energy are able to escape. They were close to the target 100 fs after the interaction but for later times they moved away, i.e. towards the EOS detector (1 mm far from the target). This is represented by the lack of red points at high energy in Fig. 4. It follows that only the high energy component of the emitted electrons is able to reach and be detected by the EOS monitor, while the low energy one is locked close to the target. These results are in agreement with measurements, consisting of detected electrons with energies larger than 6 MeV.

## Discussion

In conclusion we reported, for the first time, the dynamics of energetic electrons in short-pulse high-intensity laser matter interactions at sub-picosecond level. Our measurements provide ‘snapshots’ of the electron cloud evolution with an unprecedented resolution better than 100 fs. We have measured a significant increase in the charge and energy of the escaping electrons (corresponding to the increase in the potential barrier) for sharp structured targets. These results demonstrate the field enhancement conjecture previously predicted and can be used as a guideline in order to achieve higher energies for positively charged ions with respect to what is currently obtained through conventional laser acceleration schemes.

## Methods

### Laser and experimental area

**Figure 5:**
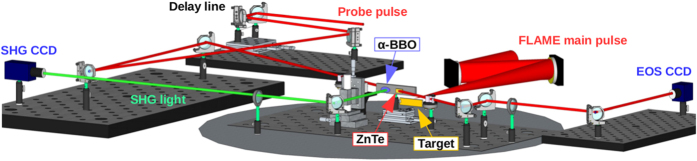
Sketch of the optical setup in the vacuum chamber. The main pulse (red line) is focused on the target by an *f*/10 parabola. The spot size on the target is measured with a microscope objective, making an image on the CCD camera (yellow line). Upstream the last multi-pass amplifier, about 10% of the main beam is split and re-compressed in order to be used as a probe for the EOS diagnostics (blue line). An *α*-cut BBO, mounted on the EOS crystal holder is used for main-probe synchronization by means of SHG, measured with a CCD camera outside the vacuum chamber (green line).

The FLAME facility consists in a 100 TW Ti:Sapphire chirped-pulse amplification (CPA) laser system delivering 35 fs, at 0.8 *μm* with energies up to 4 J at 10 Hz repetition rate. The experimental layout of the target area is shown in Fig. 5. The laser beam is focused by means of *f*/10 off-axis parabolic mirror with focal length *f* = 1 m. The focal spot is optimized using a deformable mirror, allowing to reach a focus dimension on target of the order of 25 *μm*. About 60% of the initial laser energy lie within such focal spot. The probe laser, used for the EOS diagnostics, is split before the last multi-pass amplifier and re-compressed to 35 fs duration. The laser-target interaction occurs in a high vacuum environment (10^−6^ mbar) in order to avoid self-focusing effects and reduce contaminations that could affect the experiment. The synchronization of the main and probe lasers in correspondence of the EOS crystal is obtained by means of an *α*-cut BBO crystal installed on the ZnTe holder. The time overlapping is then retrieved by using a 3 fs resolution delay-line and looking for light emission by second-harmonic generation (SHG).

### Electro-Optical Sampling

**Figure 6:**
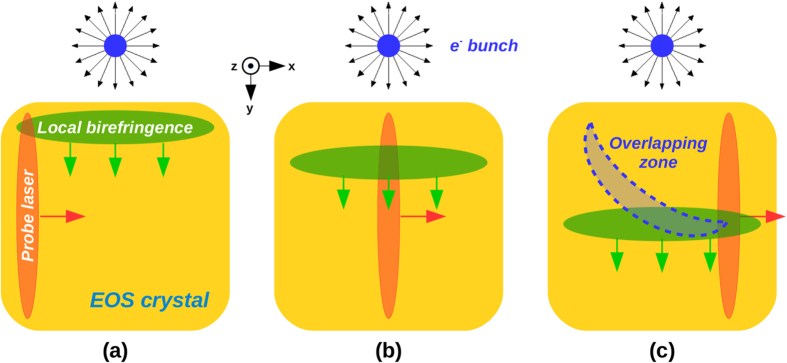
Working principle of the EOS diagnostics. (**a**) The electrons meve normally to the crystal surface (along the *z*-axis) and their Coulomb field induces a localized birefringence in the crystal. (**b**) While the electric field penetrates in the crystal, the birefringent zone shifts downwards along the *y*-axis. Simultaneously, the probe laser crosses the crystal from left and moves parallel to the *x*-axis. Its polarization is therefore rotated by means of the induced birefringence in the crystal. (**c**) The resulting signal is emitted along the blue region, i.e. where the local birefringence and the probe laser temporally overlap.

In order to retrieve the main properties of the emitted electrons (charge, energy and duration) from the measured EOS signals, a numerical simulation code has been developed in MATLAB environment. It starts calculating the transverse electric field *E*_*V*_(*t*) of a gaussian electron bunch travelling at energy *E* in vacuum at distance *r* from the ZnTe crystal. The simulation then takes into account the dispersive propagation of such field in the ZnTe crystal^30^, with thickness *d*. Being *n*(*ω*) and *κ*(*ω*) the ZnTe refractive and absorption indices in the Fourier domain^31^, the propagating field is given by
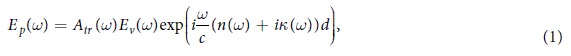
where *A*_*tr*_(*ω*) = 2/(*n*(*ω*) + *iκ*(*ω*) + 1) is the amplitude transmission coefficient. The sampling is performed by a copropagating probe laser pulse whose initial linear polarization gradually becomes elliptical due to the electro-optic effect induced by the propagating field^24^. Being *λ*_*L*_ the laser central wavelength, the overall phase delay cumulated by the probe laser at the end of the crystal is given by the convolution
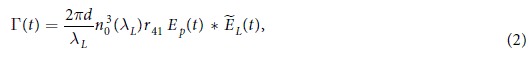
where *n*_0_ and *r*_41_ are the ZnTe optical refractive index calculated in *λ*_*L*_ and its electro-optic coefficient, respectively. 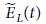 represents the normalized (dimensionless) laser electric field, also assumed to be gaussian. The process terminates by simulating the signal output (∝sin^2^(Γ(*t*)/2))) on the CCD camera. Figure 6 shows the resulting encoding process as detected by the CCD camera. The probe laser laterally enters into the crystal while the bunch, moving normally and near to it, induces a local birefringence. The bunch electric fields gradually penetrate the crystal and the localized birefringence moves vertically with a speed *c*/*n*_0_. This leads to an overlap of the probe and the birefringence along a curved path and thus a circular shape in the EOS output signals. Since the shape and strength of the detected signals depends on the bunch parameters used to calculate *E*_*v*_(*t*), by comparing the experimental data with the simulated one it is possible to extrapolate such information.

**Figure 7:**
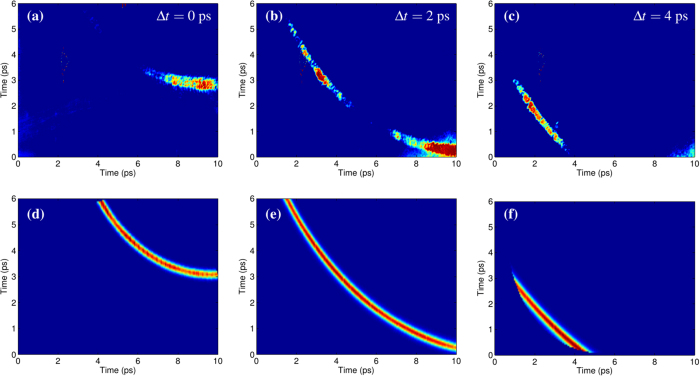
Snapshots with the wedged target. (**a–c**) Experimental measurements obtained by focusing the main laser on the edge of a wedged target at different probe laser delays (Δ*t*). The electron bunch is moving above the upper side of the crystal and normally to it. By measuring its time of flight we determined a mean energy of 15 MeV. The signal amplitude corresponds to 2.1 nC charge and the resulting bunch duration is 1 ps (rms). (**d–f**) Expected EOS signals assuming such bunch parameters. The lack of uniformity in the experimental signals, if compared with the simulated ones, is due to inhomogeneities in both the ZnTe crystal and probe laser spot.

The calibration of the EOS system been performed by focusing the Flame laser (with an intensity of about 5 × 10^18^ W cm^−2^) on the edge (about 1 *μm*-thick) of a wedged target. The resulting time-resolved signals, recorded at different delays of the probe laser, are shown in Fig. 7(a–c). The signal corresponds to a total charge of 2.1 nC of energetic electrons that have been ejected from target and traveled up to the EOS crystal in the form of ‘bunch’ with 1 ps duration and approximately energy 15 MeV energy. The reproducibility of the results is proved by looking at the three frames of Fig. 7(a–c), obtained by delaying the probe with respect to the main pulse: the signal structure remains unaltered while it moves in time (from the up-right to the down-left corner). This is confirmed in Fig. 7(d–f) where the numerical simulation of the EOS output is provided for such electron bunches. The energy information is obtained by using the EOS as a time of flight monitor^23^,^25^. Being Δ*t*_*TOF*_ the particle time of flight, we can estimate the bunch velocity as *v* = *d*/Δ*t*_*TOF*_ and its energy as *E* = *γm*_*e*_, where 
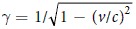
 is the relativistic Lorentz factor, *c* is the speed of light and *m*_*e*_ is the electron rest mass. Unlike conventional time-integrated spectrometric techniques, this method is able to provide energy measurements resolved in time.

## Additional Information

**How to cite this article**: Pompili, R. *et al*. Femtosecond dynamics of energetic electrons in high intensity laser-matter interactions. *Sci. Rep.* **6**, 35000; doi: 10.1038/srep35000 (2016).
